# Protocol for production of tonic CAR T cells with dasatinib

**DOI:** 10.1016/j.xpro.2024.103529

**Published:** 2024-12-30

**Authors:** Léa Rosselle, Thibault Leray, Sandy Joaquina, Benjamin Caulier, Emmet McCormack, Pascal Gelebart, Sébastien Wälchli, Else Marit Inderberg

**Affiliations:** 1 Translational Research Unit, Section for Cellular Therapy, Department of Oncology, Oslo University Hospital, Oslo, Norway; 2 Medical Faculty, University of Oslo, Oslo, Norway; 3 Institute for Cancer Research, Department of Molecular Cell Biology, Oslo University Hospital, Oslo, Norway; 4 Center for Cancer Cell Reprogramming (CanCell), Institute for Clinical Medicine, Faculty of Medicine, University of Oslo, Oslo, Norway; 5 Department of Clinical Science, Precision Oncology Research Group, University of Bergen, 5021 Bergen, Norway; 6 Centre for Pharmacy, Department of Clinical Science, University of Bergen, Bergen, Norway; 7 Centre for Cancer Biomarkers (CCBIO), University of Bergen, Bergen, Norway

**Keywords:** cell culture, cancer, health sciences, immunology

## Abstract

Chimeric antigen receptors (CARs) are synthetic molecules composed of an extracellular antigen-binding domain and an intracellular signaling domain, leading to tonic signaling and manufacturing challenges. We present a protocol for the expansion of tonic CARs by using a Food and Drug Administration (FDA)-approved kinase inhibitor, dasatinib. We report steps for T cell transduction with retrovirus, expansion and verification of CAR quality using flow cytometry, and killing assay. At only 30 nM, dasatinib improves tonic CAR T cell proliferation and quality after expansion.

For complete details on the use and execution of this protocol, please refer to Caulier et al.^1^

## Highlights

• Guidance for high CAR expression in primary T cells using retroviral transduction
• Steps for the addition of dasatinib in tonic CAR T cells culture
• Instructions for producing tonic CAR T cells of high quality, viability, and functionality

## Abstract (editor highlights)

Publisher’s note: Undertaking any experimental protocol requires adherence to local institutional guidelines for laboratory safety and ethics.

## Graphical abstract

**Figure:**
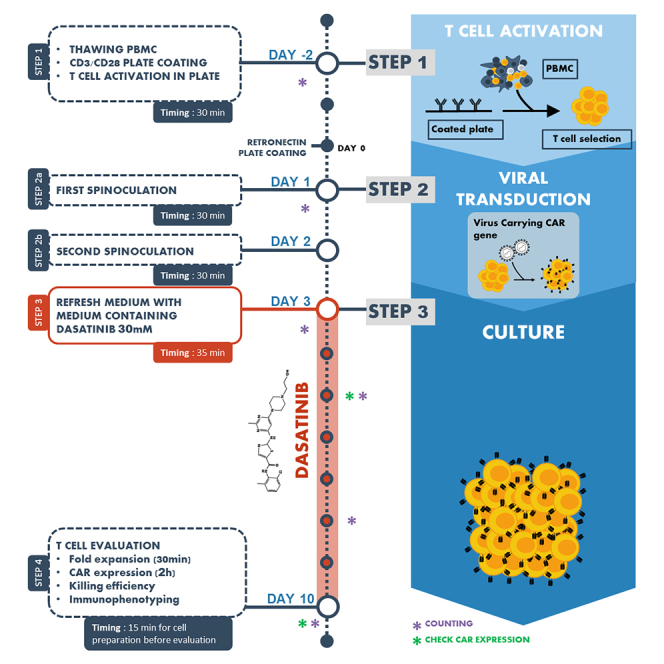

## Before you begin

The steps below describe how to culture and improve expansion of T cells expressing tonic CARs, such as CD37CAR, using dasatinib, a tyrosine kinase inhibitor. This protocol aims to provide you with a general guideline for CAR T cell production using dasatinib to improve production yield and viability. In this protocol, to ensure efficient transduction, CAR T cells are transduced with retroviral vectors. Before starting the culture process, retroviral particles must be prepared following our seminal protocol.^2^^,^^3^ Human peripheral blood mononuclear cells (PBMCs) are isolated from buffy coats by density gradient. Briefly, the blood is diluted with saline, then under layered with Lymphoprep, and centrifuged without brake. The interphase containing the PBMCs is retrieved, washed and cells are counted and either cryopreserved or used fresh. The following steps of T cell activation, retroviral transduction and CAR T cell production are described below.

**Figure 1:**
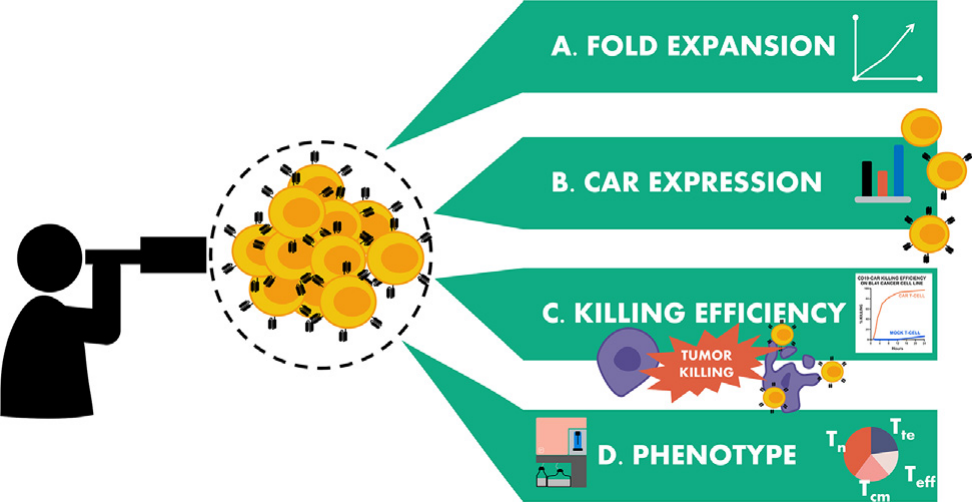
Overview of readouts: panel of relevant analyses to validate the effect of dasatinib on CAR T-cell production

**Figure 2:**
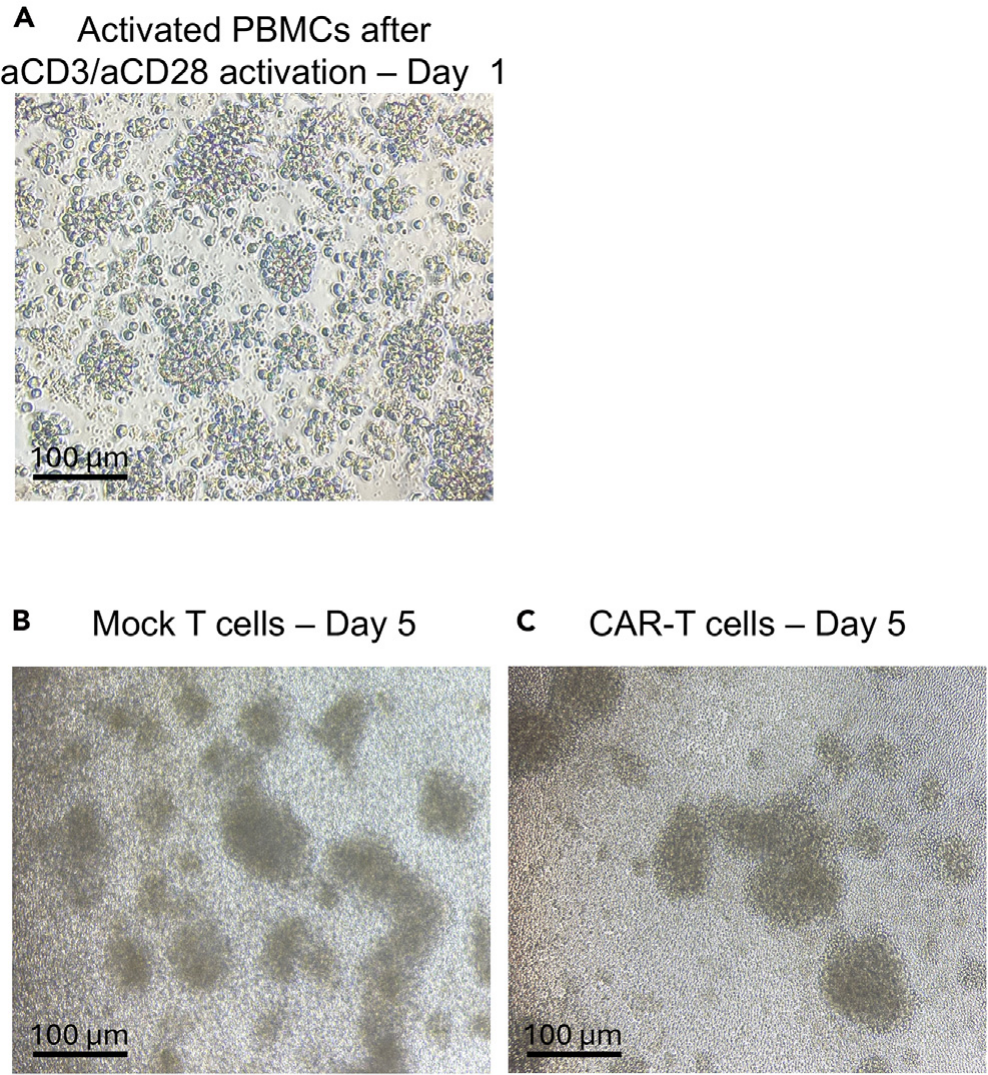
Examples of activated PBMCs and T cells in culture (A) Bright-field image of activated PBMCs after 72 h of activation with anti-CD3 and CD28 antibody stimulation (B) Bright-field image of non-transduced T cells at day 5 (C) Bright-field image of transduced T cells at day 10. The results displayed are representative of three donors.

### Institutional permission

The use of human PBMCs has been validated by the Regional Ethical Committee under the approval number 2019/121. Informed consent was obtained from all donors. Users of this protocol will need to acquire permission from their relevant institutions to use human PBMCs.

### PBMCs activation

**Timing: 15 min followed by overnight incubation (between 12 and 20 h) (for step 1)**

**Timing:2hfollowed by 3 days of incubation (for step 2)**

1. Anti-CD3/Anti-CD28 coating.
  a. Dilute anti-CD3 (1 mg/mL) and anti-CD28 (1 mg/mL) antibodies at 1:1000 in the appropriate volume of DPBS 1× and use immediately.
    **CRITICAL:** Resuspend antibodies well either by pipetting up and down or vortexing.
    ***Note:*** Prepare what you need with 1 well of margin and use immediately for proper activation of PBMC. Use the mix directly after preparation.
  b. Transfer 1 mL of antibodies and DPBS 1× mix in the appropriate number of wells needed in a 24-well cell culture plate (Corning).
  c. Leave overnight at 4°C (between 12 and 20 h).
    **CRITICAL:** Seal the lid of the plate using parafilm to avoid spillover or potential evaporation.
    ***Note:*** Alternatively, the coated plate can be incubated for 3 h at room temperature (RT) (Between 18°C and 25°C).
2. Peripheral Blood Mononuclear Cell (PBMC) activation.
  a. Thaw PBMCs and resuspend them in complete X-VIVO 15 medium (100 IU/mL recombinant IL-2 and 5% human serum (HS)).
    ***Note:*** To maximize PBMCs viability after thawing, preferably incubate thawed PBMCs at 37°C, 5% CO_2_ for 1 or 2 h in a 50 mL Falcon tube with the cap loosened. The concentration should be between 1 and 5 × 10^6^ cells/mL.
    ***Note:*** Human serum can be replaced with CTS Immune Cell Serum Replacement (SR) (Gibco) at the same concentration.
    ***Note:*** Source of PBMCs may vary. They can be isolated from Leukopaks, whole blood or buffy coat, for which numerous standard protocols by density gradient centrifugation can be used.
  b. Count PBMCs using trypan blue to assess viability and adjust the concentration to 1 × 10^6^ cells/mL in complete X-VIVO 15 medium.
  c. Remove antibody mix from the anti-CD3/anti-CD28 coated plate.
  d. Add 1 mL (1 × 10^6^ PBMCs) per well to the previously coated 24-well plate.
  e. Incubate for 72 h at 37°C, 5% CO_2_ without disturbing the cells or changing the medium.
    **CRITICAL:** Cells should not be disturbed during the first days of the activation. They can be checked under the microscope for activation (clustering). To limit movement, plates should be handled carefully.

## Key resources table

**Table:** 

REAGENT or RESOURCE	SOURCE	IDENTIFIER
**Antibodies**		

Anti-human CD3 (OKT3) antibody (1:1,000)	eBioscience, Invitrogen	Cat. No. 16-0037-85
Anti-human CD28 (CD28.6) antibody (1:1,000)	eBioscience, Invitrogen	Cat. No. 16-0288-85
Murine fragment antigen-binding-biotin antibody (1:200)	Sigma-Aldrich	Cat. No. B0529.5
G4S-AF647 antibody (1:50)	Cell Signaling Technology	Cat. No. 69782
Streptavidin-APC (1:100)	Life Technologies	Cat. No. SA1005
CD3 monoclonal antibody (SK7), Brilliant Ultra Violet 496 (1:100)	eBioscience, Invitrogen	Cat. No. 364-0036-42
CD4 monoclonal antibody (RPA-T4), eFluor 506 (1:100)	eBioscience, Invitrogen	Cat. No. 69-0049-42
CD8a monoclonal antibody (RPA-T8), Brilliant Ultra Violet 563 (1:400)	eBioscience, Invitrogen	Cat. No. 365-0088-42
CD62L (L-selectin) monoclonal antibody (MEL-14), Brilliant Ultra Violet 805 (1:100)	eBioscience, Invitrogen	Cat. No. 368-0621-82
CD197 (CCR7) monoclonal antibody (3D12), Brilliant Ultra Violet 737 (1:100)	eBioscience, Invitrogen	Cat. No. 367-1979-42
CD45RA monoclonal antibody (HI100), Brilliant Ultra Violet 395 (1:100)	eBioscience, Invitrogen	Cat. No. 363-0458-42
Brilliant Violet 570 anti-human CD45RO antibody (1:100)	BioLegend	Cat. No. 304226
CD27 monoclonal antibody (O323), APC-eFluor 780 (1:100)	eBioscience, Invitrogen	Cat. No. 47-0279-42
CD183 (CXCR3) monoclonal antibody (CEW33D), PE-eFluor 610, eBioscience (1:200)	eBioscience, Invitrogen	Cat. No. 61-1839-42
CD95 (APO-1/Fas) monoclonal antibody (DX2), PerCP-eFluor 710 (1:100)	eBioscience, Invitrogen	Cat. No. 46-0959-42
BB515 mouse anti-human CD278 (1:100)	BD Biosciences	Cat. No. 564549
CD137 (4-1BB) monoclonal antibody (4B4 (4B4-1)), PE-Cyanine7 (1:50)	eBioscience, Invitrogen	Cat. No. 25-1379-42
Pacific Blue anti-human CD69 antibody (1:100)	BioLegend	Cat. No. 310920
CD279 (PD-1) monoclonal antibody (eBioJ105 (J105)), APC (1:100)	eBioscience, Invitrogen	Cat. No. 17-2799-42
BD Pharmingen PE mouse anti-human TIGIT (1:100)	BD Pharmingen	Cat. No. 568672
Brilliant Violet 421 anti-human CD366 (Tim-3) antibody (1:100)	BioLegend	Cat. No. 345008

**Biological samples**		

Human peripheral blood mononuclear cells (PBMCs)	Healthy donors	REK vest 2012/2247

**Chemicals, peptides, and recombinant proteins**		

Recombinant human IL-2 (Proleukin)	Clinigen	Cat. No. M000276
Trypan blue solution 0.4%	Invitrogen	Cat. No. T10282
RetroNectin recombinant human fibronectin fragment	Takara Bio	Cat. No. T100B
Fetal bovine serum stock – heat inactivated	Gibco	Cat. No. 10500-064
Gentamycin	Gibco	Cat. No. 15750-037
Human serum	PAN-Biotech	Cat. No. P40-2702HI
Dasatinib	LC Laboratories	Cat. No. D-3307
XenoLight D-Luciferin potassium salt	PerkinElmer	Cat. No. PN122796
LIVE/DEAD fixable near IR (780) viability kit, for 633 nm excitation	Invitrogen	Cat. No. L34993

**Experimental models: Cell lines**		

BL-41	Leibniz Institute DSMZ-German Collection	ACC160
U937	Sigma-Aldrich Norway AS	85011440-1VL
U937 CD37 KO	In-house	Caulier et al.^1^

**Recombinant DNA**		

Luciferase-GFP plasmid	Dr. Rainer Löw	N/A
CD19CAR plasmid	In-house	N/A
CD37CAR plasmid	In-house	Caulier et al.^1^

**Software and algorithms**		

FlowJo v.10.1	FlowJo, LLC	https://www.flowjo.com/
GraphPad Prism v.9.1.0	GraphPad Software	https://www.graphpad.com/

**Other**		

Dulbecco’s phosphate-buffered saline (DPBS) 1×	Gibco	Cat. No. 14190-144
X-VIVO 15	Lonza Bioscience	Cat. No. BE02-060Q
Roswell Park Memorial Institute (RPMI) 1640	Biowest	Cat. No. L0500
24-well cell culture plate	Corning	Cat. No. 3524
24-well Nunc plate	Thermo Fisher Scientific	Cat. No. 144530
T25 culture flasks	VWR	Cat. No. 734-1712
T75 culture flasks	VWR	Cat. No. 734-2705
96-well, untreated, flat-bottomed, BRANDplates, white	VWR	Cat. No 735-2005
eBioscience Foxp3/transcription factor staining buffer set	eBioscience, Invitrogen	Cat. No. 00-5523-00
Storage plates, 96-well, MASTERBLOCK (2 mL, V-bottom, sterile)	VWR	Cat. No. 736-013 4
PerkinElmer VICTOR X4 multimode plate reader	PerkinElmer	https://www.perkinelmer.com/
BD FACSLyric clinical flow cytometry system	BD Biosciences	https://www.bdbiosciences.com/en-us/products/instruments/flow-cytometers/clinical-cell-analyzers/facslyric
Cytek Aurora 5-Lasers	Cytek	https://cytekbio.com/pages/aurora

**CRITICAL:** Dasatinib is a hazardous reagent and must be handled in a chemical foam hood. Once diluted the working stock can be handled in ordinary cell culture conditions.

## Materials and equipment

**Table:** Complete X-VIVO 15 medium

Reagent	Final concentration	Amount
X-VIVO 15	95%	950 mL
IL-2 (500K IU/mL)	0.02%	0.2 mL
Human Serum	5%	50 mL
Total	N/A	1000 mL

***Note:*** Store at 2°C–4°C, can be used for up to 2 months, if kept sterile. This storage limitation has been confirmed with the product references listed in this protocol and should be confirmed when using IL-2 from other manufacturers.

**Table:** Complete X-VIVO 15 medium containing dasatinib

Reagent	Final concentration	Amount
X-VIVO 15	95%	950 mL
IL-2 (500K IU/mL)	0.02%	0.2 mL
Human Serum	5%	50 mL
Dasatinib (1 mM)	0.003%	0.03 mL
Total	N/A	1000 mL

***Note:*** Store at 2°C–4°C, can be used for up to 2 months, if kept sterile. This storage limitation has been confirmed with the product references listed in this protocol and should be confirmed when using IL-2 from other manufacturers.

**Table:** CD3/CD28 coating

Reagent	Final concentration	Amount
CD3 antibody (stock solution at 1 mg/mL)	0.1%	0.05 mL
CD28 antibody (stock solution at 1 mg/mL)	0.1%	0.05 mL
DPBS 1× without Ca2+ and Mg2+	99.8%	50 mL
Total	N/A	50 mL

***Note:*** Prepare what you need with 1 well of margin and use immediately for proper activation of PBMC. Use the mix directly after preparation.

**Table:** FACS Buffer

Reagent	Final concentration	Amount
1× PBS without Ca2+ and Mg2+	98%	490 mL
Heat inactivated FBS	2%	10 mL
Total	N/A	500 mL

***Note:*** Store at 2°C–4°C, the buffer can be used for up to 6 months, if kept sterile.

## Step-by-step method details

Activation: day −2.

Transduction: day +1 and +2.

Addition of dasatinib: day +3.

T cell evaluation: day +10. (Figure 1)

***Note:*** T cells are normally harvested on day 10 after first spinoculation but can be cultured for up to 12 days before being used in experiments or frozen.

### T cell transduction with retrovirus

**Timing:2days,including preparation and incubation**

**Timing: 15 min followed by overnight incubation with RetroNectin (between 12 and 20 h) (for step 1)**

**Timing: 10 min followed by 30 min incubation (for step 2)**

**Timing: 30 min (for step 3)**

**Timing: 30 min followed by 1 h of centrifugation and 24 h incubation (for step 4)**

**Timing: 30 min followed by 1 h of centrifugation and 24 h incubation (for step 5)**

After 72 h of activation, the T cells are actively dividing and ready to be efficiently transduced with viral particles. Clusters should be observed after PBMCs activation, as shown in Figure 2A. The mitotic state allows the integration of the construct into the genome for its stable expression. At this step, the cells do not yet express the CAR molecules. Thus, as there is no problem of autonomous tonic signaling, dasatinib is not needed.

1. RetroNectin Coating (on Day 0 of the timeline).
  a. Thaw RetroNectin (Diluted in DPBS at 50 μg/mL and stored at −20°C) at RT.
  b. Coat non-treated 24-well plates with 300 μL per well.
    **CRITICAL:** For an efficient transduction, it is important to coat the entire surface of the well with RetroNectin. Tilt the plate to cover the entire well and avoid bubbles.
    ***Note:*** A non-treated plate can be used as the coating with RetroNectin will allow cell adhesion and contact with the viral particles.
  c. Incubate overnight at 4°C (between 12 and 20 h).
    **CRITICAL:** Seal the lid of the plate using parafilm to avoid spillover or potential evaporation.
    ***Note:*** Alternatively, the coated plate can be incubated for 3 h at room temperature (RT) (between 18°C and 25°C).
2. Blocking of unspecific epitopes (On Day 1 of the timeline).
  a. Remove the RetroNectin solution.
  b. Add 1 mL of blocking solution (sterile PBS with 2% FBS final) in each coated well.
    **CRITICAL:** The liquid should be added gently into the coated plates to avoid disturbing the coating.
  c. Incubate for 30 min at RT.
  d. Remove the blocking solution just before adding the virus (Step 3-b).
    **CRITICAL:** Remove the blocking solution just before the addition of the viral particles to avoid drying.
    ***Note:*** A minimum of one well per construct is needed and one control well without virus particles must be done as a negative control (mock). Calculate the number of wells that you need according to your experiment. For example, if you have 3 donors and 2 constructs that you want to test in duplicate, you will need to coat 18 wells.
3. Activated T cells preparation (On Day 1 of the timeline).
  a. Check the activated T cells under a bright field microscope (Cells obtained after 72 h of culture from the “Before to begin” step 2) and the color of the medium.
    **CRITICAL:** The T cells have to be in a proliferative phase for efficient retroviral transduction. Check the aspect of the culture: The well should not contain adherent cells, red blood cells (non-nucleated, flat disk shape) or platelets (Small, non-nucleated, granular cells) and T cells should look activated and healthy, clusters of cells should be observed as depicted in Figure 2A. Check the viability and count the cells as an indicator of health and proliferation. The cells should have a viability of at least 80% after activation to obtain good transduction efficiency.
  b. Harvest the cells in a tube.
  c. Centrifuge at 500 × *g* for 5 min and discard the supernatant.
  d. Resuspend in 1 mL of complete X-VIVO 15 medium.
  e. Count the cells.
  f. Add the suitable volume of complete medium to obtain 0.6 × 10^6^ cells/mL.
4. First Spinoculation (On Day 1 of the timeline).
  a. Centrifuge the viral supernatant for 5 min at 700 × *g* at RT to spin down the cell debris of your virus production.
    **CRITICAL:** Viral supernatant may be contaminated with HEK293T cells from virus production protocol.^3^ Excess cell debris can also fix to the RetroNectin and reduce the binding of the virus that will lead to reduced transduction efficacy.
  b. Pre-warm the centrifuge to 32°C before performing the spinoculation of the plate with the T cell/virus mix.
  c. Add 500 μL of the viral supernatant production or 500 μL of medium to the control condition.
    **CRITICAL:** Gently add the liquid into the coated wells to avoid coating damages.
    ***Note:*** For the transduction control (Mock), add 500 μL of the medium used for the virus production.^2^
  d. Add 500 μL of the T cell suspension at 0.6 × 10^6^ cells/mL to achieve 0.3 × 10^6^ cells in each well.
    **CRITICAL:** Viral particles must be added first as they have to bind to the RetroNectin for a better interaction with the T cells.
  e. Centrifuge the plate at 750 × *g* for 60 min at 32°C to improve the interaction between virus and RetroNectin.
  f. Check the cells under a bright field microscope. They should be equally distributed in the well and be at the bottom of the well.
  g. Incubate at 37°C with 5% CO_2_.
5. Second Spinoculation (On Day 2 of the timeline).
  ***Note:*** To further increase the transduction efficiency and transgene expression, a second spinoculation is performed.
  a. Check the cells under a bright field microscope and the color of the medium.
  b. Centrifuge the plate for 5 min at 750 × *g* to spin all the cells down into the well.
  c. Carefully remove 500 μL of supernatant.
    **CRITICAL:** Do not mix the cells and avoid touching the bottom of the well to avoid cell aspiration. With the centrifugation, the cells stick to the RetroNectin and will not be aspirated by careful pipetting.
  d. Add 500 μL of the viral supernatant or 500 μL of medium to the control condition.
  e. Centrifuge the plate at 750 × *g* for 60 min at 32°C.
  f. Check the cells under a bright field microscope.
  g. Leave the plate at 37°C with 5% CO_2_.

### Expansion with dasatinib

**Timing:7days (from day 3 to day 10 of the timeline)**

**Timing: 5 min (for step 6)**

**Timing: 30 min followed by the 6 days incubation (for step 7)**

**Timing: 15 min (for step 9)**

As the T cell culture volume contains half the volume of viral particle supernatant (generally DMEM with 1% FBS), it is important to refresh the medium for optimal culture conditions. At this stage 30 nM dasatinib is added to the complete X-VIVO 15 culture medium. This step is performed 24 h post-transduction, corresponding to Day 3 of the Timeline. The concentration of dasatinib has been established according to previous publications on CAR T cell culture with dasatinib.^4^

6. Dasatinib medium preparation (On day 3 of the timeline).
  a. Add 30 μL of dasatinib 1 mM stock solution to 1 L of complete X-VIVO 15 medium (final dasatinib concentration is 30 nM).
  b. Mix the medium by rotation of the bottle to homogenize the dasatinib.

**CRITICAL:** Dasatinib is non-soluble in water and has to be dissolved in DMSO. The final concentration of DMSO in the culture medium should not exceed 0.1%. Reduce the concentration of your stock solution if needed.

***Note:*** Store at 2°C–4°C, can be used for up to 2 months, if kept sterile. This storage limitation has been confirmed with the product references listed in this protocol and should be confirmed when using IL-2 from other manufacturers.

***Note:*** In the present protocol, X-VIVO 15 supplemented with HS and 100 IU/mL of IL-2 is used. Other sera, medium supplements and cytokines could be used. Preliminary tests should be performed to validate their efficiency with dasatinib and the CAR of interest.

7. Cell washing (On day 3 of the timeline, the day after the second spinoculation).
  a. Check the aspect of cells under a bright field microscope and the color of the medium.
  b. Harvest the cells into a 5 mL tube.
  c. Centrifuge at 500 × *g* for 5 min and discard the supernatant.
  d. Resuspend the cells in 1 mL of fresh complete X-VIVO 15 medium containing dasatinib.
  e. Count and resuspend at 0.6 × 10^6^ cells/mL.
    ***Note:*** Following anti-CD3 and anti-CD28 activation, T cells are strongly activated and expand well. For this reason, seeding the cells at 0.6 × 10^6^ cells/mL is recommended. If the cells do not seem sufficiently activated, the density can be increased to 1 × 10^6^ cells/mL at this step.
  f. Redistribute the cells into a new 24-well plate treated for cell culture.
  g. Incubate at 37°C with 5% CO_2_.
8. Six days of T cell expansion (From day 4 to day 10 of the timeline).
  a. Check the aspect of cells every 1–2 days under a bright-field microscope and the color of the medium.
    ***Note:*** T cells form clusters during expansion which is a sign of stimulation and proliferation as seen on Figures 2B or 2C.
  b. When the T cells reach confluency and the medium turns orange/yellow, add the suitable volume (generally 1:1) of complete X-VIVO 15 medium containing dasatinib at 30 nM.
    **CRITICAL:** The medium should not become yellow as it is an indicator of acidic pH. With the production of CAR T cells, cells can be more activated and the medium becomes more yellow, probably due to the release of high amounts of metabolites due to high metabolic activity. In case this happens, add more medium (1:1 ratio with old medium) in the conditions with a higher T cell density or with an orange/yellow color. This is usually done every 2 days, but some CAR T cell cultures may require more frequent medium addition.
  c. Control CAR expression by flow cytometry on day 5 as described in part C of this protocol.
9. T cell preparation for evaluation (On day 10 of the timeline).
  a. Check the aspect of cells under a bright field microscope and the color of the medium (Figures 2B or 2C).
  b. Count and repeat CAR expression analysis by flow cytometry (as described in part C).
  c. Wash cells to remove the remaining dasatinib before any type of functional assay.

**CRITICAL:** Dasatinib is used during T cell expansion to improve the yield and viability of T cells expressing tonic CAR. At the end of the expansion on day 10, the cells are washed in complete X-VIVO 15 medium to remove dasatinib. As dasatinib may impair CAR signaling, all efficacy testing of the CAR T cells also has to be done without its presence. 24 h after washing, a complete reversion of the dasatinib effect was observed.

For clinical manufacturing, the CAR T cells are washed before cryopreservation and infusion into the patient.

**Figure 3:**
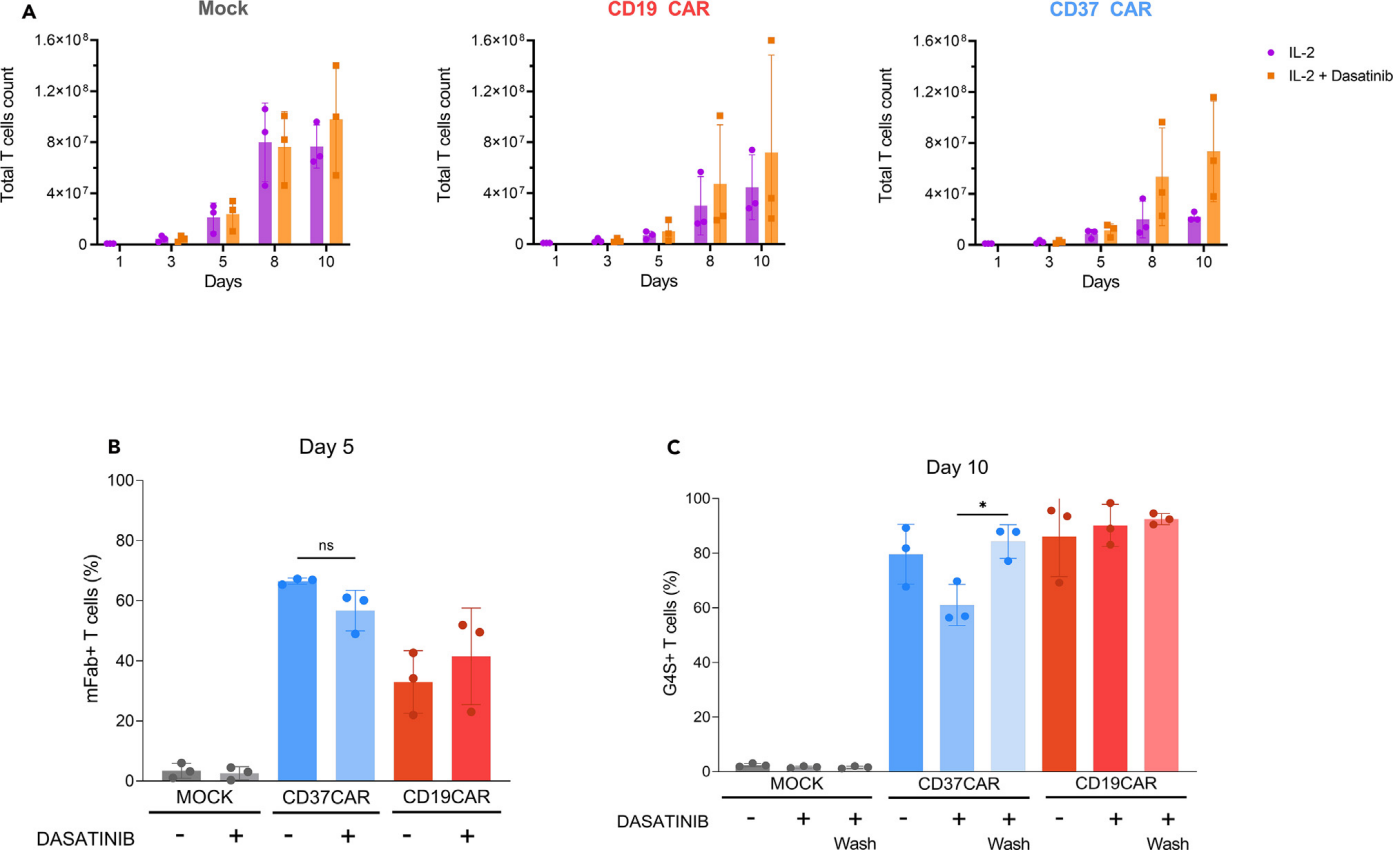
Expansion of T cells in culture and characterization of CAR expression (A) Expansion shown as total cell count normalized to the untreated condition of T cell donors bearing the CAR constructs (*n* = 3) for 10 days post-transduction. Percentage of CAR expression in T cells at day 5 (B) detected using an anti-murine fragment antigen-binding (mFAb) antibody for CD19 and CD37CAR and at day 10 (C) using an anti-G4S linker antibody. Unpaired t-test was performed. Comparisons with CD37CAR not treated with dasatinib are displayed, ∗ *p*-value < 0.05, ns = not significant. Error bars represent standard deviation.

***Note:*** By starting with 3 × 10^5^ cells, a fold expansion up to 100× is expected (Figure 3). The expansion is generally dependent on your starting material (donor and origin: Leukopaks, healthy donor or patient-derived cells). T cells from patients may expand more poorly depending on cancer stage and previous treatments.^5^

### CAR expression

**Timing:2h**

At day 5 and 10, CAR surface expression in primary T cells is verified using flow cytometry. A check of CAR expression at the membrane on day 5 is critical to ensure proper expression of the CAR to continue with further expansion and experiments. In this protocol, two different types of staining were performed, one using an anti-murine fragment antigen-binding-biotin (mFab) antibody at day 5 and an anti-G4S-AF647 antibody at day 10 to display different ways of CAR detection.

10. Harvest 200 μL of cell culture and transfer into a polystyrene tube suitable for flow cytometry.

***Note:*** At this step, counting cells is not required to perform the rest of the staining process.

11. Wash each sample in FACS Buffer by centrifuging for 5 min at 500 × *g*.
12. Stain cells with anti-mFab antibody at a 1:200 dilution for 20 min at RT, protected from light.

***Note:*** Stain cells with anti-G4S-AF647 antibody at 1:50 dilution for 15 min at room temperature, protected from light.

13. Wash samples once with FACS Buffer.

***Note:*** When G4S staining is performed, secondary staining is unnecessary. Proceed directly to step 7.

14. Perform a secondary staining with streptavidin-APC at a 1:100 dilution for 10 min at RT, protected from light.
15. Wash samples with FACS Buffer.
16. Resuspend cells in 250 μL of FACS Buffer.

***Note:*** After staining, cells can be stored at 4°C, protected from light for a couple of hours before being assessed by flow cytometry.

## Expected outcomes

CAR T cell therapy is very promising for hard-to-cure cancers, but “live” drugs create manufacturing challenges that need to be addressed. Due to their structure, CAR molecules may contain different combinations of stimulatory domains. Basal signaling can therefore occur with varying strength depending on the CAR type. The T cell receptor (TCR) naturally exhibits weak ligand-independent tonic signaling, important for cell homeostasis.^6^ Thus, by adding similar intracellular domains to CAR molecules, this constant physiological signal is also observed in CAR T cells and is a sign of efficiency. However, in some cases, CARs exhibit strong autonomous signaling that leads to overstimulation and can impact their expansion through poor viability, low amount of cells and exhaustion.^7^^,^^8^ Many actors in the signaling pathway could be modulated to reduce this uncontrolled excitation by the use of selective drugs such as dasatinib.

Dasatinib has been described as a drug used to control tonic signal in CAR T cells.^9^^,^^10^^,^^11^ Its reversible effect on CAR T cells also makes it a good candidate for supplement in culture. Several studies have shown that in the case of CAR T cells, the lymphocyte-specific protein kinase (Lck) is the main target of dasatinib and leads to the inhibition of Zeta-chain-associated protein kinase 70 (ZAP70) and CD3 zeta phosphorylation.^9^^,^^12^^,^^13^ However, as these two tyrosine kinases are also involved in TCR signaling, an early addition of dasatinib could impair anti-CD3/anti-CD28 activation. To prevent this effect, dasatinib was added 5 days after activation of PBMCs.

**Figure 4:**
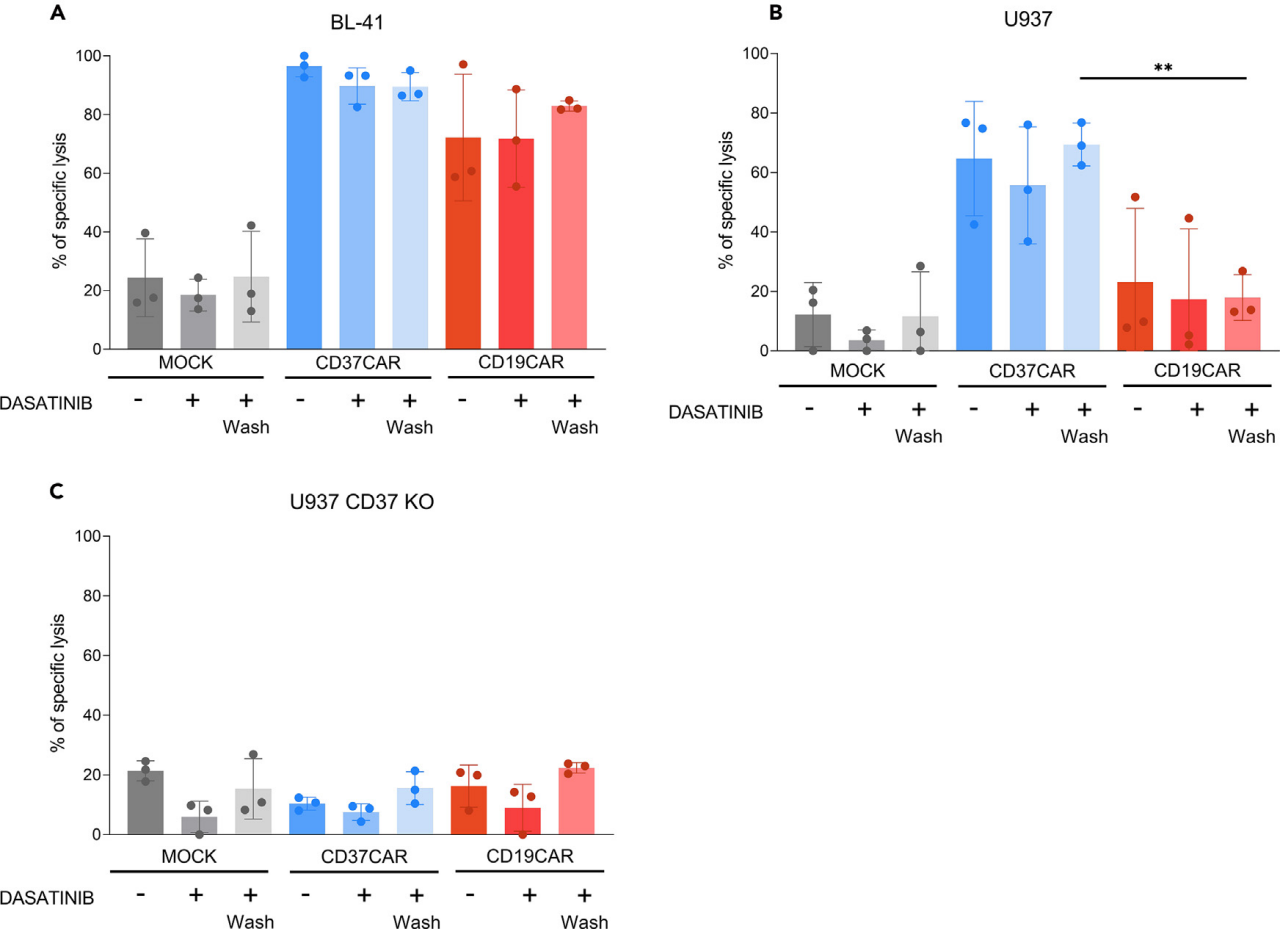
Evaluation of transduced T-cell functionality using cytotoxicity assay Specific lysis of T cells incubated for 8 h with (A) BL-41, (B) U-937 and (C) U-937 CD37KO. The E:T ratio displayed here is 5:1 (*n* = 3 donors). Cell lines were genetically modified to express luciferase. The luminescence signal is then assessed, and specific lysis is calculated using the following formula: 100∗(Spontaneous luminescence-experimental)/ luminescence-maximum lysis). Paired t-tests were performed between conditions. Only comparisons between CD19CAR and CD37CAR conditions are shown. ∗∗ *p* value < 0.05. Error bars represent standard deviation.

**Figure 5:**
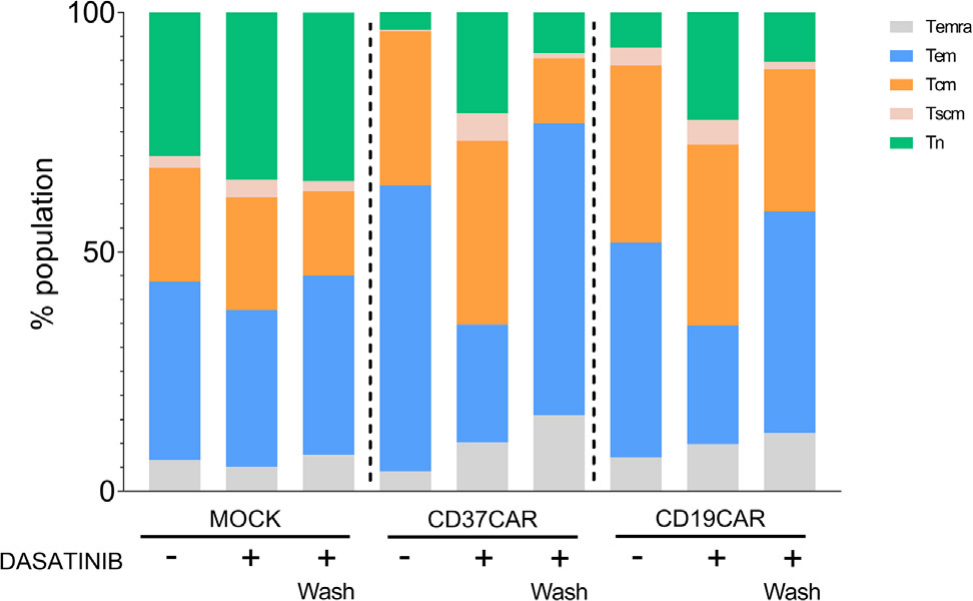
Characterization of the CAR T cell subsets using spectral flow cytometry Percentage of T cell subsets on Total CD3+ cells 10 days after transduction and cultured without dasatinib (−), with dasatinib (+) or with dasatinib and washed 24 h before the analysis (+ Wash) (*n* = 3 donors).

**Figure 6:**
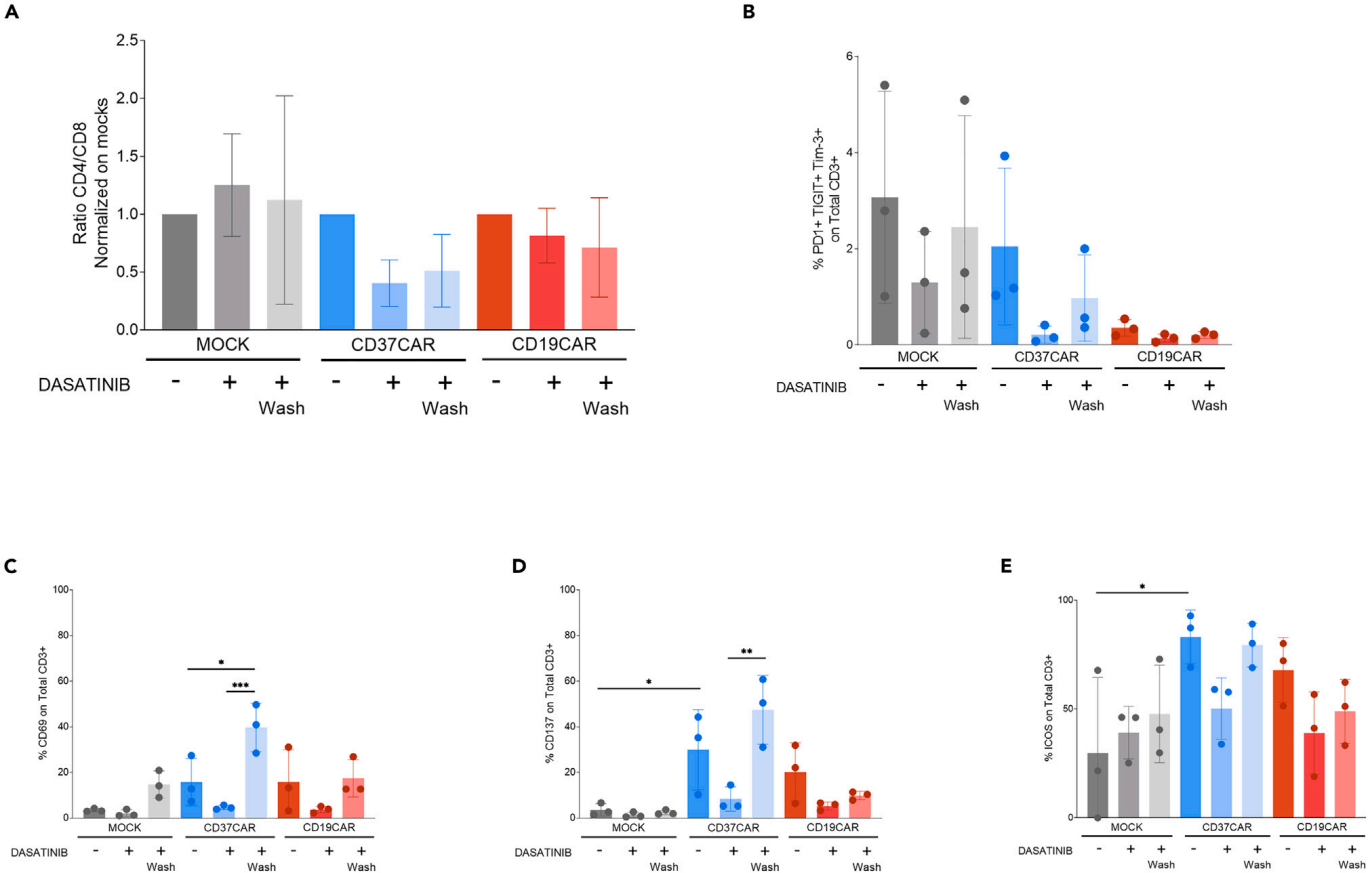
Modulation of T cell function with dasatinib (A) CD4/CD8 ratio, (B) exhaustion marker expression, (C, D, E) activation marker expression of CAR T cells 10 days after transduction and cultured without dasatinib (−), with dasatinib (+) or with dasatinib and washed 24 h before the analysis (+ Wash). Ordinary one-way ANOVA followed by Tukey’s multiple comparisons tests was performed. Comparisons versus CD37CAR are displayed, ∗∗∗*p* < 0.0001, ns = not significant. (*n* = 3 donors). Error bars represent standard deviation.

Here we aimed to improve the production of CAR T cells with poor ability to expand due to tonic signaling. The protocol was successfully used for the establishment of our recently published CD37CAR^1^ and is reproducible across donors (Figures 3, 4, 5, and 6). In the example presented below, the validation was done by comparing the CD37CAR with the non-transduced T cells (Mock) and a non-tonic CAR, CD19CAR. To validate the efficiency of the CAR, several functional *in vitro* assays have to be performed at the end of the culture. For instance, our routine analyses are fold expansion, CAR expression detection by flow cytometry, cytotoxicity against target cell lines, and phenotyping (Figure 1). We expect a higher fold expansion of tonic CAR T cells with dasatinib and no impact of dasatinib on CAR expression. We also predict a similar killing capacity with or without dasatinib. Finally we suppose that culture with dasatinib will block T cell differentiation in non-renewable T cell subsets (effector T cells) and decrease activation and exhaustion markers.

### Expansion

Throughout the whole culture of primary T cells, fold expansion was assessed by counting cells using Trypan Blue 0.04% on Kova slides. Cells were counted on days 1, 3, 5, 7, and 10 in the different conditions to follow the expansion.

According to Figure 3A, dasatinib does not have any impact on the expansion of mock T cells and CD19CAR T cells for two out of three donors. These data might suggest the lack of toxicity of this component, confirming its potential as a T cell culture component. Interestingly, it seems that dasatinib positively impacts the expansion of CD37CAR T cells compared to their non-treated counterparts with a strong trend towards increased expansion for three out of three donors.

These data confirm previous observations made by Caulier et al.^1^ where they showed that dasatinib may be useful to improve CD37CAR T cell expansion *in vitro*. This effect might be observed because this tyrosine kinase inhibitor may limit the CD37CAR T cell tonic signaling. Therefore, it could impair the exhaustion of such T cells in the culture and favor a better expansion in such experimental settings.

### Evaluating car expression

During CAR T cell culture, it is critical to verify CAR expression during and at the end of the whole expansion. In this paper, CAR expression was characterized by flow cytometry using an antibody targeting the CAR molecules. For this purpose, the single-chain variable fragment (scFv) of the CAR or a linker part of the CAR can be the target depending on the construct that is used. The staining protocol used in this paper is described above in section C.

As observed in Figures 3B and 3C, there is no significant influence of dasatinib on the percentage of positive CAR T cells for both CD19CAR and CD37CAR T cells. Staining at day 5 and at day 10 are performed using different methods of detection, thus cannot be directly compared between the two time points. According to Caulier et al.^1^ CAR expression usually remains stable throughout the whole culture. According to Figure 3C, only the removal of dasatinib 24 h before analysis shows a significant increase of CD37CAR expression at the membrane. This result could be explained by the rescue of CAR recycling at the membrane in the absence of dasatinib in culture media.

### Cytotoxicity assay

After CAR T cell expansion and validation of CAR expression, it is crucial to confirm the functionality of expanded CAR T cells. In this paper, the functionality of transduced T cells was assessed by cytotoxicity assay. To estimate *in vitro* cytotoxicity, a bioluminescence-based killing assay protocol was performed as previously described in Caulier et al.^1^

In brief, target cells selected for this assay were retrovirally transduced to express the luciferase reporter gene. The target cells were then treated with luciferin substrate and co-cultured at 5:1 effector to target ratio (E:T ratio) for 24 h at 37°C, 5% CO_2_. Luminescence was measured every 2 h using a multimodal plate reader able to read luminescence. The 8-h time point is displayed in Figure 4. The percentage of specific lysis was calculated using the following formula: 100∗(Spontaneous luminescence-experimental)/(luminescence-maximum lysis).

As shown in Figure 4, this assay demonstrates the potency and specificity of CD37CAR against tested cell lines. As expected according to results observed by Caulier et al.,^1^ Figure 4B demonstrates specific lysis of the pro-monocytic U937 cell line by CD37CAR T cells only treated or not with dasatinib. Together these data show that dasatinib does not impact overall killing capacity of CD37CAR T cells against CD37 positive cell lines BL-41 or U937. The BL-41 lymphoma cell line is also CD19 positive.

Due to previous observations and signaling impairing properties of dasatinib, a decrease in the percentage of specific lysis in the presence of dasatinib may be expected. The absence of a significant effect of dasatinib in these experimental settings could be explained by the low concentration of dasatinib used in the culture media. Such low concentration could be sufficient to prevent tonic signaling but it may not have any impact on killing properties of CD37CAR T cells.^4^

Functionality assessment of T cells is critical to demonstrate the potency of developed CAR. It is also interesting to focus on the phenotype of such transduced T cells and how culture conditions can impact the overall phenotype of your population that may explain potential differences in responses.

### Phenotyping

The phenotype of CAR T cells was evaluated by surface marker staining and spectral flow cytometry to examine whether the phenotype was differentially impacted by dasatinib or not. For analysis, the T cell subsets were determined according to the expression of CD62L and CD45RA as follow: Naïve T cells (Tn): CD45RA + CD62L+, Central memory T cell (Tcm): CD45RA- CD62L+, Effector memory T cells (Tem): CD45RA- CD62L-, Terminally differentiated T cells (Temra): CD45RA + CD62L-. The stem-like T cells (Tscm) were selected with the markers CD45RA + CD62L + CD27+ CXCR3+ CD95+ (Figure S1). The spectral flow panel also includes T cell markers (CD3, CD4, and CD8), activation markers (CD69, CD137, ICOS) and exhaustion markers (PD-1, Tim-3, and TIGIT) allowing us to determine the influence of dasatinib on T cell function.

The phenotype of the tonic CD37CAR included more differentiated effector memory cells due to increased stimulation during the expansion (Figure 5). However, this subset does not retain a high proliferative capacity and overstimulation leads to early cell death explaining the low viability observed during expansion leading to a lower expansion rate. By cultivating the CAR T cells with dasatinib, the phenotype became more similar to that of mock T cells and included a higher percentage of Tn which have a higher renewal capacity. Interestingly, dasatinib did not seem to have any impact on Mock T cell phenotype. In addition, with a non-tonic CAR (CD19CAR), we observed a slight increase of naïve phenotype, but this had no impact on fold expansion or cytotoxicity. Thus, the positive influence of dasatinib observed on the quality of tonic CAR T cell cultures could be due to the observed phenotype switch.

Interestingly, only 24 h after the removal of dasatinib from the culture, we observed a reestablishment of the distribution of the different T cell subsets similar to cultures without dasatinib, confirming what has been described in the literature.^4^^,^^14^

Regarding the additional markers, we observed an impact on the ratio of CD4:CD8 by dasatinib for CD37CAR T cells (Figure 6A). Dasatinib seemed to increase the proportion of CD8 T cells which could be an advantage for the cytotoxic efficiency of the CAR T cells. The CD4:CD8 ratio was not impacted for Mock or CD19CAR T cells.

Dasatinib has been described to reduce T cell exhaustion linked to tonic signaling.^14^ To confirm this, we assessed the expression of exhaustion markers PD-1, Tim-3 and TIGIT after 10 days. For Mock and CD37CAR, the expression of exhaustion markers decreased in presence of dasatinib. In contrast, after 24 h without dasatinib, an increase in the expression of such markers was observed (Figure 6B). Interestingly, the exhaustion marker expression of the CD37CAR T cells after washing did not reach the level of the culture without dasatinib, which could explain the improved efficiency *in vitro* (Figure 4) and *in vivo*.^1^ Due to inter-donor variations, there was a trend, but no significant differences.

The impact of dasatinib on activation markers seemed to be more important (Figures 6C, 6D, and 6E). Indeed, both with CD37CAR and CD19CAR, the expression of CD69, CD137 and ICOS was reduced in the presence of dasatinib and the effect was reversed after 24 h without dasatinib. As *in vivo* injection of the dasatinib-cultured T cells showed effective long-term protection,^1^ this re-increase of activation markers associated with an increase in the proportion of effector cells could be one of the boosters of CAR efficacy and lead to more efficient killing of cancer cells. In addition, the higher proportion of Tscm observed in Figure 5, although modest, in the final product of the dasatinib culture could increase the persistence *in vivo* and thus provide enhanced protection.^15^

These results are consistent with several studies that have shown that dasatinib inhibits T cell differentiation and exhaustion.^4^^,^^9^^,^^10^^,^^11^^,^^14^ Hebbar et al.^4^ studied the tonicity of a GRP78-CAR T cells and concluded that dasatinib enhances the effector function of GRP78-CAR T cells both *in vitro* and *in vivo* by driving differentiation and exhaustion. In this study, dasatinib was removed just before *in vitro* analysis, but *in vivo* assays showed a persistence of the injected T cells over 80 days.^4^ Interestingly, the authors concluded that CAR surface expression was inhibited by dasatinib leading to a more efficient cell expansion that might be due to the absence of the extracellular recognition part. In our case, the surface expression of the CAR seemed slightly modulated by dasatinib, involving a real modulation of the tonic signal.

### Conclusion

**Figure 7:**
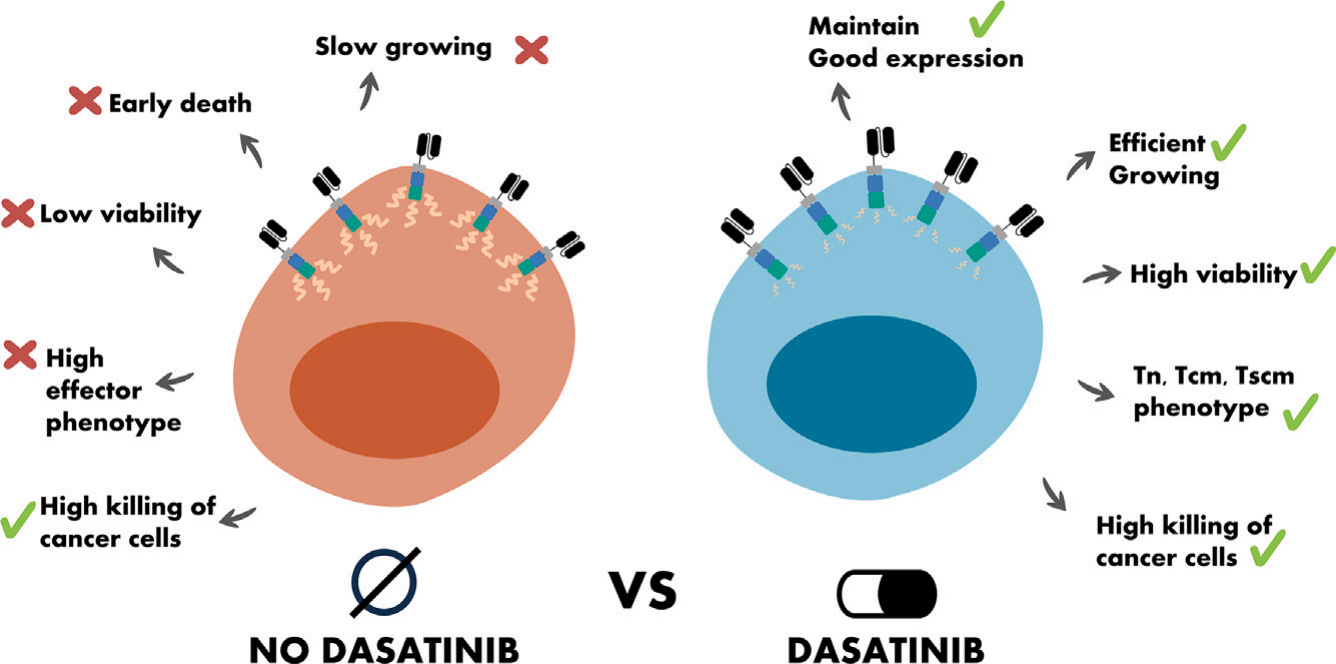
Expected outcomes

By following the present protocol and performing different characterizations and functional assays, the production of tonic CAR T cells can be highly improved and greatly facilitated by dasatinib in accordance with previously published studies (Figure 7).

## Limitations

This protocol describes a method for the culture of tonic CAR T cells with a multityrosine kinase inhibitor, dasatinib. Therefore, it assumed that the culture issues were induced by autonomous tonic signaling due to one or several tyrosine kinases involved in CAR signaling causing uncontrolled activation. Here the protocol was tested with CD37CAR (tonic) and CD19CAR (non-tonic) and only improved CD37CAR T cell manufacturing, whereas CD19CAR T cells remained unaffected. If the culture issues are due to another phenomenon this protocol could be inefficient.

## Troubleshooting

### Problem 1

Low CAR Expression.

### Potential solutions

• In case of low Viral Production Quality (See before you begin): Verify the quality of the viral particles by evaluating the transduction efficiency using a control cell line, such as Jurkat cells, before applying it to primary T cells. If frozen viral supernatant is used, ensure it is thawed just before use to maintain viral integrity. Fresh viral supernatant can be kept at 4°C for a few days only. For long term storage, the viral supernatant should be kept at −80°C in small aliquots to avoid freeze/thaw cycles.
• In case of CAR Detection Method being variable (See CAR expression): Confirm that the method used for CAR detection (e.g., flow cytometry) is optimized and that the antibodies used are specific and sensitive enough to detect CAR expression. For example, the extracellular part of murine CAR molecules can be detected through the staining with an antibody binding the F(ab')2/Fab portion mouse IgG, i.e., the single-chain variable fragments (scFvs). CARs used in this protocol are designed with murine scFvs. Thus, a Murine Fragment antigen-binding-biotin antibody was used for detection of CARs at day 5. An alternative, if the CAR contains a G4S linker used to connect the VH and VL domain of the scFV, is to use an antibody specific for this linker. Ensure that antibodies are properly stored according to the manufacturer’s instructions to prevent loss of activity or fluorescence, especially avoiding prolonged exposure to room temperature. Use different detection methods to ensure the efficiency of the detection.
• In case of suspicion of poor RetroNectin Coating (See RetroNectin coating): Verify that T cells attach and spread homogeneously on the whole surface of the well. Ensure proper coating of the transduction plate with RetroNectin solution. Store the stock solution of RetroNectin at −20°C and thaw it just before use. If the coating duration exceeds 3 h, keep the coated plate at 4°C to maintain its functionality.

### Problem 2

Poor Activation of PBMCs with anti-CD3 and anti-CD28 antibodies.

### Potential solutions

• In case of low cell viability (See before you begin): Assess the quality of PBMCs before transduction. Healthy PBMCs should form small clusters upon activation. Proper thawing of PBMCs is crucial to ensure high viability; allow them to rest for 1–2 h post-thawing to recover from the stress of freezing. To perform an efficient transduction, activated PBMCs viability should be above 80%. Ensure a sufficient initial seeding density of 1 × 10^6^ cells/mL for optimal activation.
• In case of bad coating quality (See before you begin): Inadequate or uneven plate coating with antibodies can lead to poor activation. Ensure that the coating solution covers the entire surface of the well evenly and ensure thorough mixing of antibodies in PBS to achieve a homogenous coating solution. Poor resuspension may result in inconsistent activation across wells. Ensure that antibodies are properly stored under appropriate conditions to preserve their activity.

### Problem 3

Donor Variability.

### Potential solutions

Minimize variability by standardizing the protocol for all donors. Use consistent activation, transduction, and culture conditions.

### Problem 4

Low Viability of Tonic CAR T Cells with dasatinib Addition (See Dasatinib medium preparation).

### Potential solutions

• In case of non-suitable dasatinib Storage: Dasatinib is less stable in solution and must be stored at a high concentration at −20°C. Prepare fresh stock solutions as needed and store aliquots properly to maintain stability.
• In case of incorrect mixing and precipitation: Ensure dasatinib is thoroughly mixed into the culture medium. Inspect the medium for any precipitate that may indicate poor quality of dasatinib or wrong dissolution. Dasatinib should be first dissolved in DMSO before being added to the medium. Make a new stock solution and new medium in case of non-homogenized dasatinib.
• In case of wrong dosage: If low viability persists, consider titrating the concentration of dasatinib. Increase the concentration and monitor cell viability and functionality.

### Problem 5

Dasatinib impairs CAR T Cell Function.

### Potential solutions

• In case of high dosage (See **Dasatinib medium preparation)**: If dasatinib suppresses CAR T cell function by inhibiting T cell receptor signaling too strongly, consider titrating the dasatinib by reducing the concentration. It is also possible to decrease the dose towards the end of the expansion to allow partial recovery of CAR T cell function.
• In case of longer recovery Time (See **T cell preparation for evaluation)**: After dasatinib removal, allow sufficient time for CAR T cells to recover before performing analysis. Perform a washout step and incubate cells for several hours or overnight. The effect of dasatinib is reversible so the functionality of the T cell should be recovered before performing any functional assays.

### Problem 6

Insufficient Cell Expansion (See materials and equipment).

### Potential solutions

• In case of non-optimal cytokine support: Verify that the culture medium contains sufficient cytokine support (e.g., IL-2) for T cell proliferation. Ensure the cytokines are active and used at the recommended concentrations. Confirm the bioactivity of cytokines, especially if they are expressed in mass concentration (e.g., ng/mL). The activity of cytokines can vary between batches and suppliers.

### Problem 7

High Levels of Non-T Cell Contamination.

### Potential solutions

• In case of non-suitable medium (See materials and equipment): Use T cell-specific culture media to select T cells and reduce the growth of non-T cell populations.
• In case of non-suitable cytokine selection (See materials and equipment): Adjust the cytokines in the culture medium to favor T cell growth over non-T cell contaminants.
• If contamination persists (See before you begin): use a T cell isolation kit to purify the T cell population, such as magnetic bead-based negative selection.

## Resource availability

### Lead contact

Further information and requests for resources and reagents should be directed to and will be fulfilled by the lead contact, Else Marit Inderberg, (Else.Marit.Inderberg@rr-research.no).

### Technical contact

Questions about the technical specifics of performing the protocol should be directed to the technical contact, Léa Rosselle, (lea.rosselle@rr-research.no).

### Materials availability

This manuscript did not generate new unique reagents.

### Data and code availability

This study did not generate data sets code.

## Acknowledgments

This study was partially supported by the Research Council of Norway NFR KSP-2021 CellFit project (326811) to E.M.I. and NFR 337468, 350181, 351914, and 391915 to S.W. S.W. also received partial support from the Norwegian Health Authority South-East (2024080). L.R. is a postdoctoral fellow supported by the NFR KSP-2021 CellFit project (326811). T.L. is a PhD student supported by the Marie Skłodowska-Curie Actions program MELOMANES funded by the Horizon 2020 program of the European Union (101073025). B.C. was a postdoctoral fellow of the Norwegian Cancer Society (208012) and also supported by the Scientia Fellow II—Marie Skłodowska-Curie Actions program (801133). S.J. was a PhD student supported by era-net EURONANOMED-3 NAN-4-TUM (310531) and is now supported by a grant from the Barnekreftforeningen and Jonathans Minnefond (PERCAP, 230004). E.M. and P.G. were partially supported by grants from the Research Council of Norway (326300) and the Norwegian Cancer Society (223171).

We thank the Flow Cytometry Core Facility at the Institute for Cancer Research (Oslo University Hospital). We are grateful to the Radiumhospitalets Legater for their support.

## Author contributions

Conceptualization, S.W. and E.M.I.; methodology, L.R., T.L., B.C., and S.J.; validation, L.R., T.L., B.C., S.J., E.M., and P.G.; formal analysis, L.R. and T.L.; investigation, L.R., T.L., B.C., and S.J.; data curation, L.R., T.L., B.C., and S.J.; writing – original draft, L.R. and T.L.; writing – review and editing, B.C., S.J., E.M., P.G., S.W., and E.M.I.; visualization, L.R. and T.L.; supervision and funding acquisition, S.W. and E.M.I.

## Declaration of interests

The CD37CAR construct has been patented (WO2017118745A1), and S.W. and E.M.I. are listed among the inventors.

## Supplemental information

Document S1. Figure S1, related to step 9 and Figure 5
