# Supplementary figures and images for: Protocol for production of tonic CAR T cells with dasatinib
Source: STAR Protoc. 2024 Dec 30;6(1):103529. doi: 10.1016/j.xpro.2024.103529 (PMC11750262; doi:10.1016/j.xpro.2024.103529)

## Supplementary data

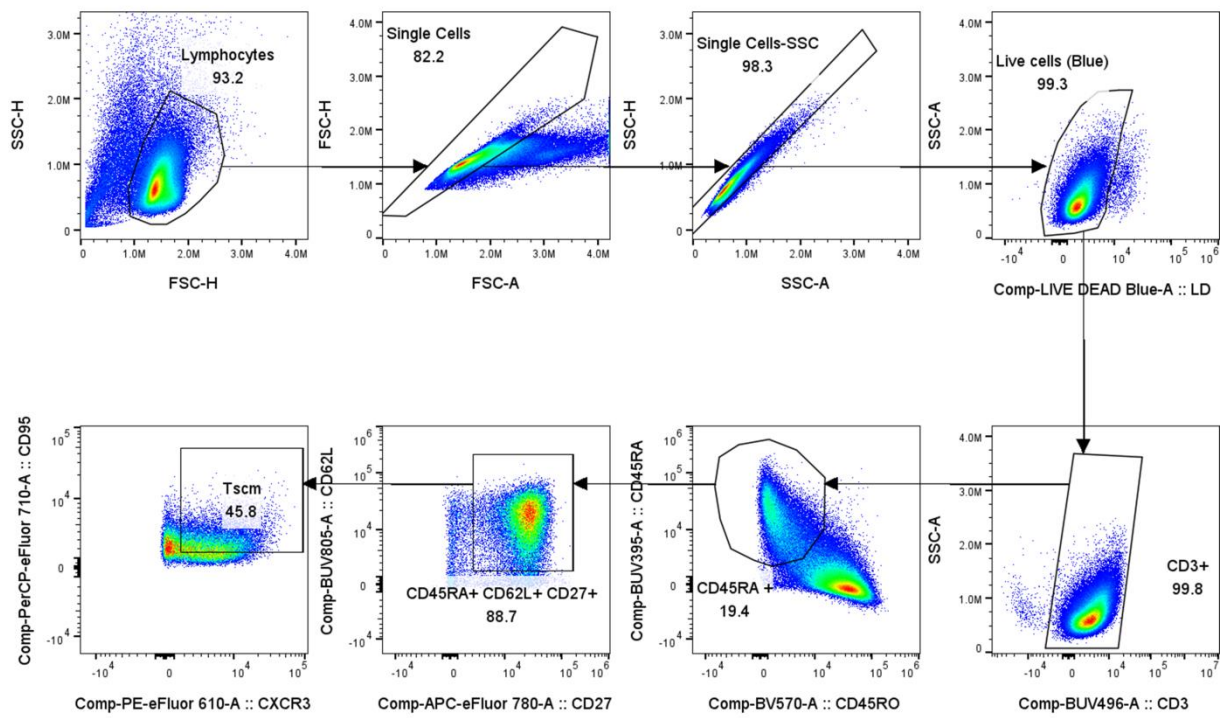

**Figure S1:** Gating strategy of the Tscm subsets (CD45RA+ CD62L+ CD27+ CXCR3+ CD95+).

Supplement: Document S1. Figure S1, related to step 9 and Figure 5 [file mmc1.pdf]
